# Evaluation of autophagy inducers in epithelial cells carrying the ΔF508 mutation of the cystic fibrosis transmembrane conductance regulator CFTR

**DOI:** 10.1038/s41419-017-0235-9

**Published:** 2018-02-07

**Authors:** Shaoyi Zhang, Gautier Stoll, José Manuel Bravo San Pedro, Valentina Sica, Allan Sauvat, Florine Obrist, Oliver Kepp, Yousheng Li, Luigi Maiuri, Naoufal Zamzami, Guido Kroemer

**Affiliations:** 1 Faculty of Medicine, University of Paris Sud-Saclay, Kremlin-Bicêtre, France; 2 Metabolomics and Cell Biology Platforms, Gustave Roussy Cancer Campus, Villejuif, France; 3 Institut National de la Santé et de la Recherche Médicale UMRS1138, Equipe 11 labellisée Ligue Nationale contre le Cancer, Centre de Recherche des Cordeliers, Paris, France; 4 Sorbonne Paris Cité, Université Paris Descartes, Paris, France; 5 Gustave Roussy Comprehensive Cancer Center, Villejuif, France; 6 Université Pierre et Marie Curie, Paris, France; 7 Department of General Surgery, Shanghai Ninth People’s Hospital, Shanghai JiaoTong University School of Medicine, Shanghai, China; 8 European Institute for Research in Cystic Fibrosis, Division of Genetics and Cell Biology, San Raffaele Scientific Institute, Milan, Italy; 9 SCDU of Pediatrics, Department of Health Sciences, University of Piemonte Orientale, Novara, Italy; 10 Pôle de Biologie, Hôpital Européen Georges Pompidou, APsupp-HP, Paris, France; 11 Department of Women’s and Children’s Health, Karolinska University Hospital, Stockholm, Sweden

## Abstract

Cystic Fibrosis (CF) due to the ΔF508 mutation of cystic fibrosis transmembrane conductance regulator (CFTR) can be treated with a combination of cysteamine and Epigallocatechin gallate (EGCG). Since ECGC is not a clinically approved drug, we attempted to identify other compounds that might favourably interact with cysteamine to induce autophagy and thus rescuing the function of ΔF508 CFTR as a chloride channel in the plasma membrane. For this, we screened a compound library composed by chemically diverse autophagy inducers for their ability to enhance autophagic flux in the presence of cysteamine. We identified the antiarrhythmic Ca^2+^ channel blocker amiodarone, as an FDA-approved drug having the property to cooperate with cysteamine to stimulate autophagy in an additive manner. Amiodarone promoted the re-expression of ΔF508 CFTR protein in the plasma membrane of respiratory epithelial cells. Hence, amiodarone might be yet another compound for the etiological therapy of CF in patients bearing the ΔF508 CFTR mutation.

## Introduction

Cystic Fibrosis (CF) is the most frequent monogenetic lethal disease in human with a worldwide incidence of approximately 1:3500^1^. This autosomal recessive disease occurring results from loss-of-function mutations in the gene coding for the cystic fibrosis transmembrane conductance regulator (CFTR), a 1480-amino acid protein that acts as a cyclic adenosine monophosphate-gated chloride channel at the plasma membrane of different cells, mostly epithelial cells and macrophages^2–4^. Defective CFTR function causes reduced epithelial chloride transport and bicarbonate secretion coupled to chronic progressive lung disease with accumulation of viscous mucus, chronic inflammation, and bacterial infection^5–8^. Defective CFTR function also compromises the capacity of macrophages to clear bacteria^9–11^. CF can be caused by ~2000 different CFTR mutations, although there is one single, highly prevalent mutation that accounts for ~85% of CF cases, consisting in the deletion of phenylalanine in position F508 (ΔF508)^12–14^. This mutation affects the stability and turnover of the CFTR protein, ultimately causing its depletion from the plasma membrane and hence the loss of its function^15–19^.

Thus far, the therapy of CF patients with the ΔF508 CFTR mutation is mostly symptomatic, consisting in nutritional interventions, inhalations, physiotherapy, as well as anti-inflammatory and antibiotic treatments^20–22^. More recently, a combination of molecules able to directly target the mutated CFTR to the plasma membrane (correctors) and molecules that improve its ion channel transport (potentiators) have been FDA- and EMA-approved for the treatment of patients homozygous for the ΔF508 CFTR^23^. In addition, alternative strategies aiming at targeting the cellular environment and proteostasis networks in which the ΔF508 CFTR protein is synthesized, traffics and turned over have been explored in two recent clinical trials in patients bearing misfolded CFTR mutants either in homozygous or compound heterozygous form. This has been achieved by a novel combination therapy consisting in the sequential administration of the transglutaminase-2 inhibitor cysteamine and the green tea flavonoid Epigallocatechin gallate (EGCG). Indeed, this combination therapy can be considered as an etiological approach because children receiving this treatment recover CFTR function, as assessed by so-called sweat test that measures the capacity of the cholinergic agent pilocarpine to stimulate sodium chloride secretion by sudoriparous glands of the skin^24–26^. Normally, CF patients manifest an abnormally high salt content in the sweat due to the failure of the cells in the sweat duct to reabsorb salts^6–8^. However, after sequential treatment with cysteamine and EGCG, this laboratory parameter declines almost to normal levels indicating the restoration of CFTR function^27,28^. Signs in favour of such restoration have also been obtained in freshly isolated brushed nasal epithelial cells. In such cells, the so-called band C, which corresponds to glycosylated, plasma membrane-sessile mature CFTR protein is reduced in CF patients as compared to controls, and again cysteamine plus EGCG normalized this function^28,29^.

The mode of action of the combination treatment apparently relies on the induction of autophagy. Thus, cysteamine plus EGCG can stimulate autophagic flux in vitro, in cultured respiratory epithelia from human origin, by inhibiting the activity of TG2 which can target the master player of the autophagosome formation, Beclin1, and dislodge the phosphatidylinositol 3-kinase catalytic subunit type 3 (PIK3C3) away from the endoplasmic reticulum (ER)^27,28^. Depletion of the essential autophagy gene products ATG5 or Beclin1, as well as addition of pharmacological inhibitors of phosphatidylinositol 3-kinase catalytic subunit type 3 (PIK3C3), prevents the positive effect of the combination treatment on CFTR expression and function in vitro^27,28^. Similarly, mice bearing a knock-in mutation of their *Cftr* gene that resembles that of human ΔF508 CFTR can be treated with cysteamine plus EGCG to recover the function of the mutated CFTR protein both in lungs and gut. However, the drug combination loses its capacity to restore CFTR function in mice that lack one allele of the gene coding for Beclin 1 (*Bcln1*^+/−^) and that are partially autophagy-deficient^28^. Altogether, these results support the idea that the restoration of ΔF508 CFTR function by cysteamine plus EGCG is indeed mediated through the induction of autophagy^30^.

Less is known through which mechanisms EGCG can synergize with cysteamine. Indeed, besides the ability of EGCG to inhibit the activity of CK2 that contributes to the fragmentation of ΔF508 CFTR, EGCG can also inhibit the acetyltransferase activity of EP300, an autophagy-repressive enzyme, thereby stimulating autophagy^31–34^, and also causes protein cross-linking^35–37^. Moreover, in contrast to cysteamine, which is clinically approved for the treatment of cysteinosis^38–40^, EGCG has not (yet) the status of an FDA or EMA approved drug. Driven by these considerations, we launched a research project to identify pharmacological compounds that might replace EGCG with respect to autophagy induction and restoration of ΔF508 CFTR function. For this, we screened compound libraries with the scope of discovering drugs that synergize with cysteamine to stimulate autophagy.

## Results

### Autophagy induction by the combined treatment with cysteamine and EGCG

**Fig. 1:**
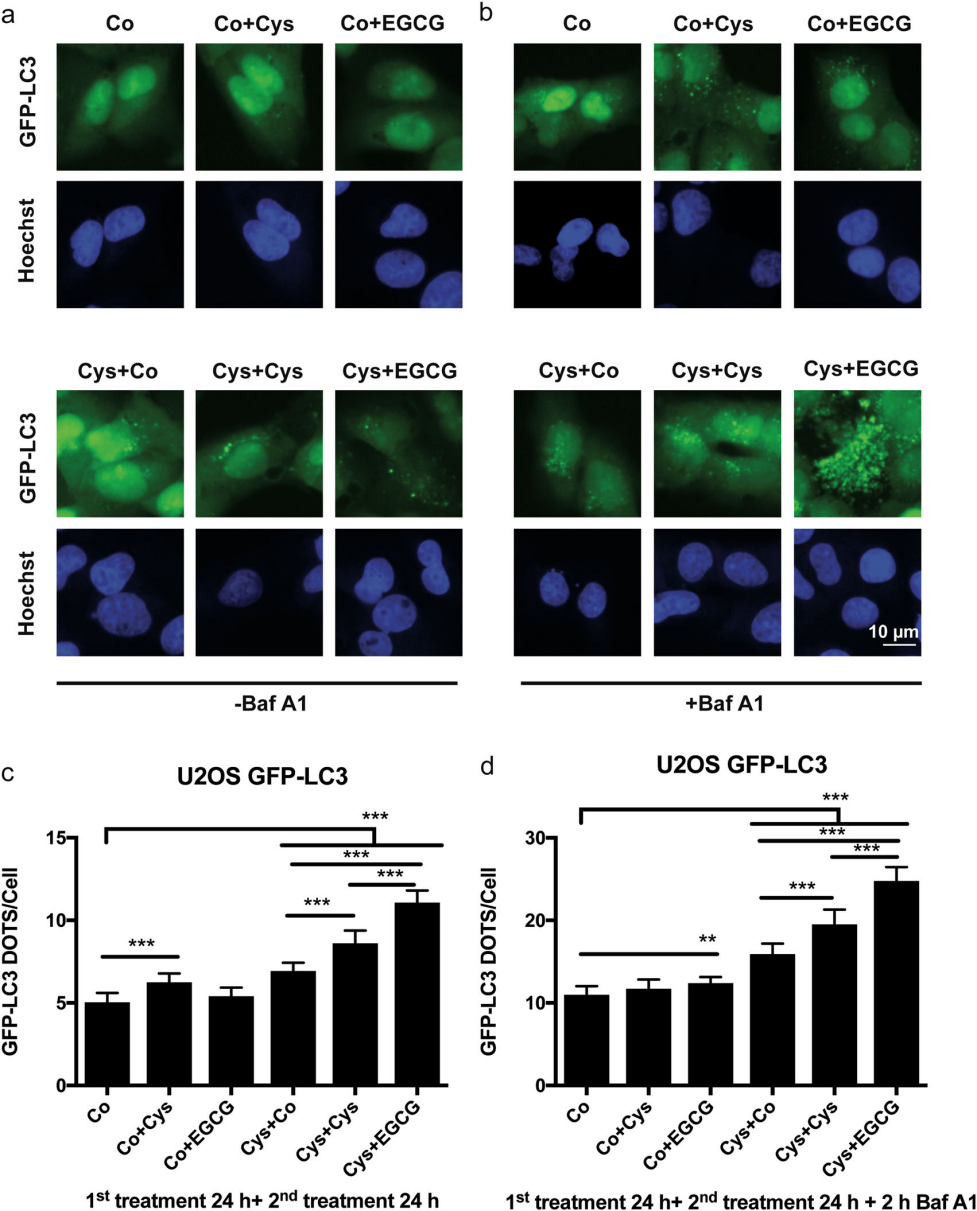
Effect of Cysteamine and Epigallocatechin Gallate (EGCG) in stimulating autophagy. **a** Representative images of U2OS cells stably expressing GFP-LC3. Upper panel: the cells were treated with complete medium (Co), cysteamine (Co+Cys, 500 μM), EGCG (Co+EGCG, 160 μM) for 24 h. Lower panel: the cells were pre-treated 24 h with cysteamine (500 μM) then washout with PBS, following additionally incubation for 24 h with complete medium (Cys+Co), cysteamine (Cys+Cys, 500 μM), or EGCG (Cys+EGCG, 160 μM). **b** In order to assess the autophagic flux, the cells were treated, as described in (**a**) in the presence of bafilomycin A1 (Baf A1, 100 nM), added 2 h before the end of each experimental condition. **c**, **d** GFP-LC3 dots quantification per cell of the experimental conditions shown in (**a**) and (**b**), respectively. Data are expressed as means ± SEM of at least three independent experiments (**p < 0.01, ***p < 0.0001, compared to untreated cells, Co). Scale bars equal 10 μm

**Fig. 2:**
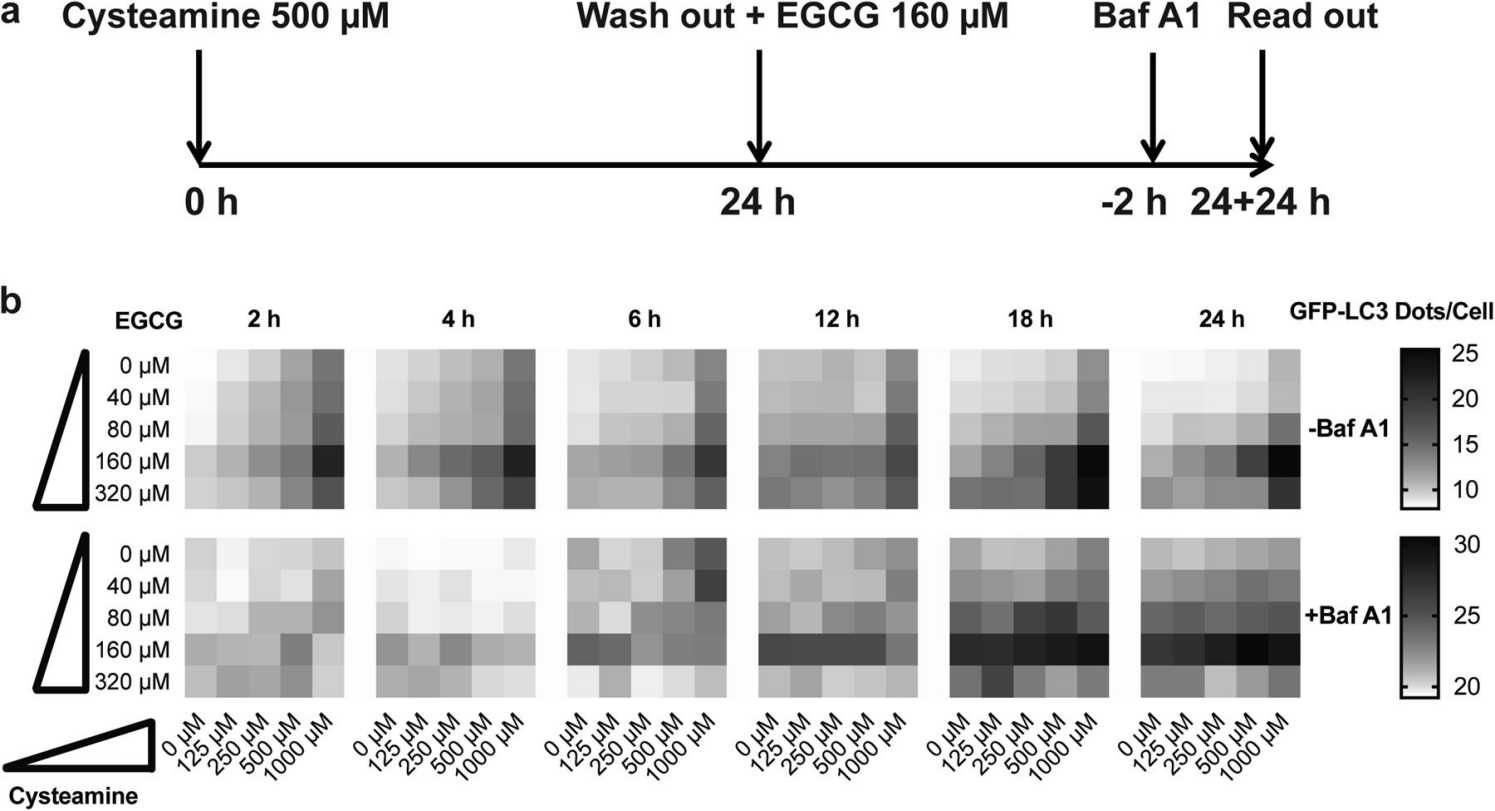
Kinetic induction of autophagy by the Cysteamine and Epigallocatechin Gallate (EGCG) co-treatment. **a** Schematic representation of the sequential treatment of cysteamine (500 μM) and EGCG (160 μM) for the cellular autophagic assessment by a high content screening microscopy. **b** U2OS cells stably expressing GFP-LC3 were seeded into a 384 well plate, the day after, cysteamine was added to each well; after 24 h of incubation, cells were washed with PBS and incubated with EGCG. Bafilomycin A1 (Baf A1, 100 nM) was added 2 h before the end of the experiment. Chessboard graph summarises the pharmacokinetic additive effect of pre-treatment of cysteamine in 5 different concentrations (0, 125, 250, 500, and 1000 μM) following by PBS washout and incubation with EGCG in 5 different concentrations (0, 40, 80, 160, and 320 μM) from 2 to 24 h. The grey scale intensity of the graph indicates the numbers of the GFP-LC3 dots per cell

**Fig. 3:**
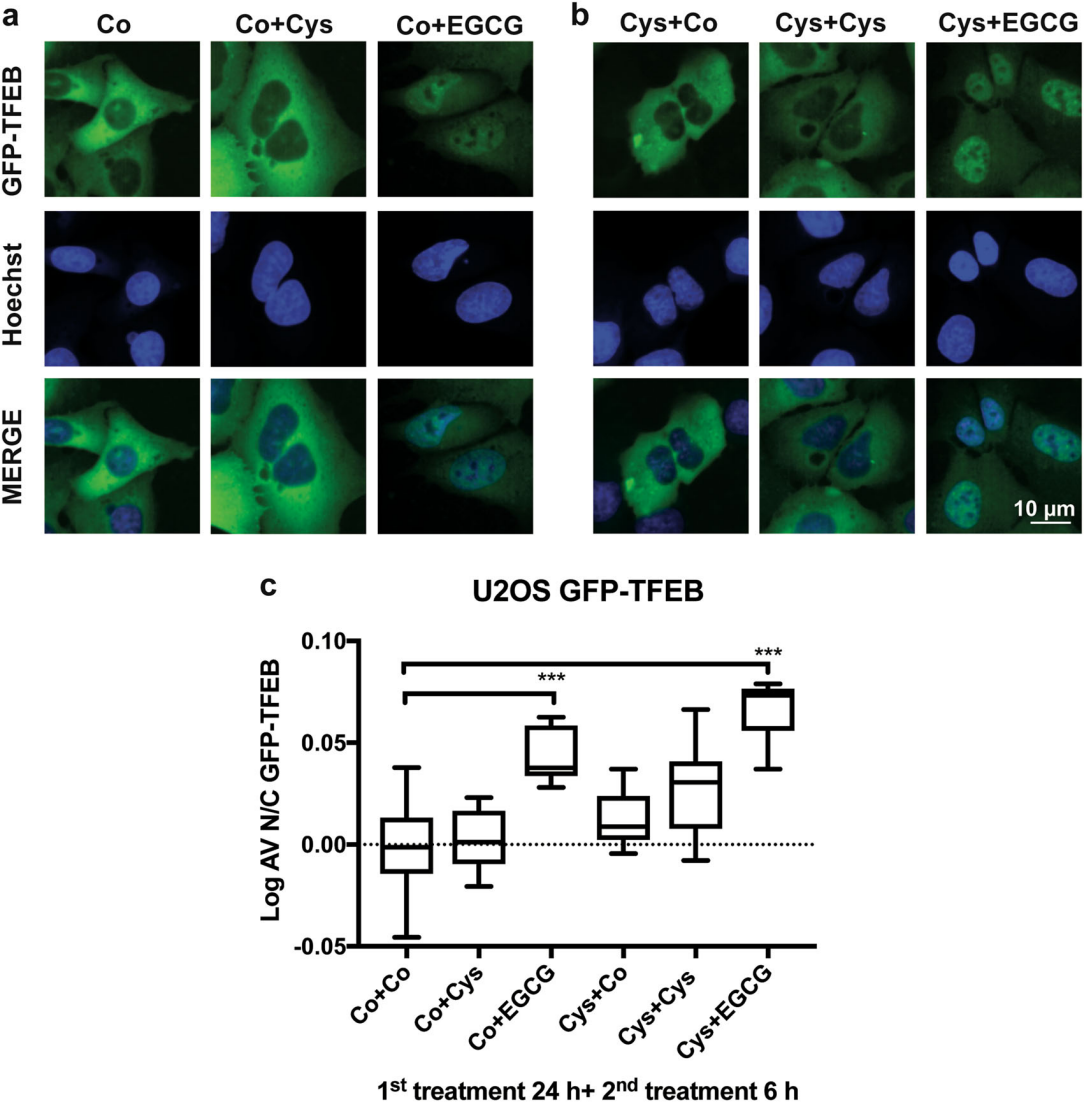
Effect of Cysteamine and Epigallocatechin Gallate (EGCG) on the transcription factor EB (TFEB) translocation from cytoplasm to nucleus. **a** Representative images of U2OS cells stably expressing GFP-TFEB treated with complete medium (Co), cysteamine (Co+Cys, 500 μM) or EGCG (Co+EGCG, 160 μM). **b** the cells were pre-treated for 24 h with cysteamine (500 μM), afterward washed with PBS, and then treated for 6 h with complete medium (Cys+Co), cysteamine (Cys+Cys, 500 μM) or EGCG (Cys+EGCG, 160 μM). Scale bars equal 10 μM. **c** Data are expressed as means ± SD (*n* = 3, ***p < 0.001 compared to untreated cells) and demonstrate the average ratio between GFP-TFEB florescence intensity in the nuclear vs. the cytoplasm (Log AV N/C GFP-TFEB indicates the normalisation value between the average GFP intensity in the nuclear vs. the cytoplasm)

Autophagy is often measured by following the subcellular distribution of a fusion protein composed by green fluorescent protein (GFP) in the N-terminus and microtubule-associated proteins 1A/1B light chain 3B (MAP1LC3B, best known as LC3) in the C-terminus^41–43^. This GFP-LC3 fusion protein is usually diffuse in the cytosol, yet translocates to the membranes of autophagosomes and autolysosomes^42,43^. To evaluate the direct impact of cysteamine and EGCG treatment on autophagy flux, we took advantage of human osteosarcoma U2OS cells stably expressing a GFP-LC3 chimera^44,45^. The cells were kept in the absence (Co) or presence of 500 µM cysteamine for 24 h, then washed and cultured for another 24 h with medium alone (Co), 500 µM cysteamine or 160 µM EGCG (Fig. 1a, c). This protocol was inspired by observations that were made previously in epithelial cells showing that the effects of cysteamine on CFTR stability was maintained several hours after cells were washed^27,28,33,46^. The absolute number of GFP-LC3 puncta was quantified by fluorescence microscopy analysis in GFP-LC3 U2OS cells treated as previously described or in presence of Baf A1, added during the last 2 h of the experiment to measure autophagic flux (Fig. 1b, d). These data show that the sequential treatment with cysteamine plus EGCG yielded a higher number of GFP-LC3 dots per cell than either of the two agents alone. Next, we modified this protocol (Fig. 2a), while keeping the first treatment phase constant (24 h), though with variable concentrations of cysteamine (125 µM to 1 mM), followed by incubation with EGCG (40–320 µM) for an interval ranging from 2 to 24 h. This experiment corroborated an additive effect of cysteamine and EGCG on GFP-LC3 dots accumulation, especially at 18–24 h of incubation, both in the absence and in the presence of Baf A1 added during the last 2 h of the experiment to measure autophagic flux (Fig. 2b). Protracted autophagic turnover requires transcriptional activation of pro-autophagic genes by transcription factor EB (TFEB)^47^. TFEB is normally inactive in the cytoplasm and translocates into the nucleus when it becomes activated^48,49^. When U2OS cells stably expressing a GFP-TFEB fusion protein were treated following the standard protocol (Fig. 2a), nuclear localization of TFEB associated fluorescence was only observed in response to treatment with EGCG or the combination of EGCG plus cysteamine, not cysteamine alone (Fig. 3).

Altogether, these results point out the additive effect of cysteamine and EGCG to stimulate autophagic flux as they provide a mechanistic explanation for this additive effect.

### Screening of autophagy inducers for combination effects with cysteamine

**Fig. 4:**
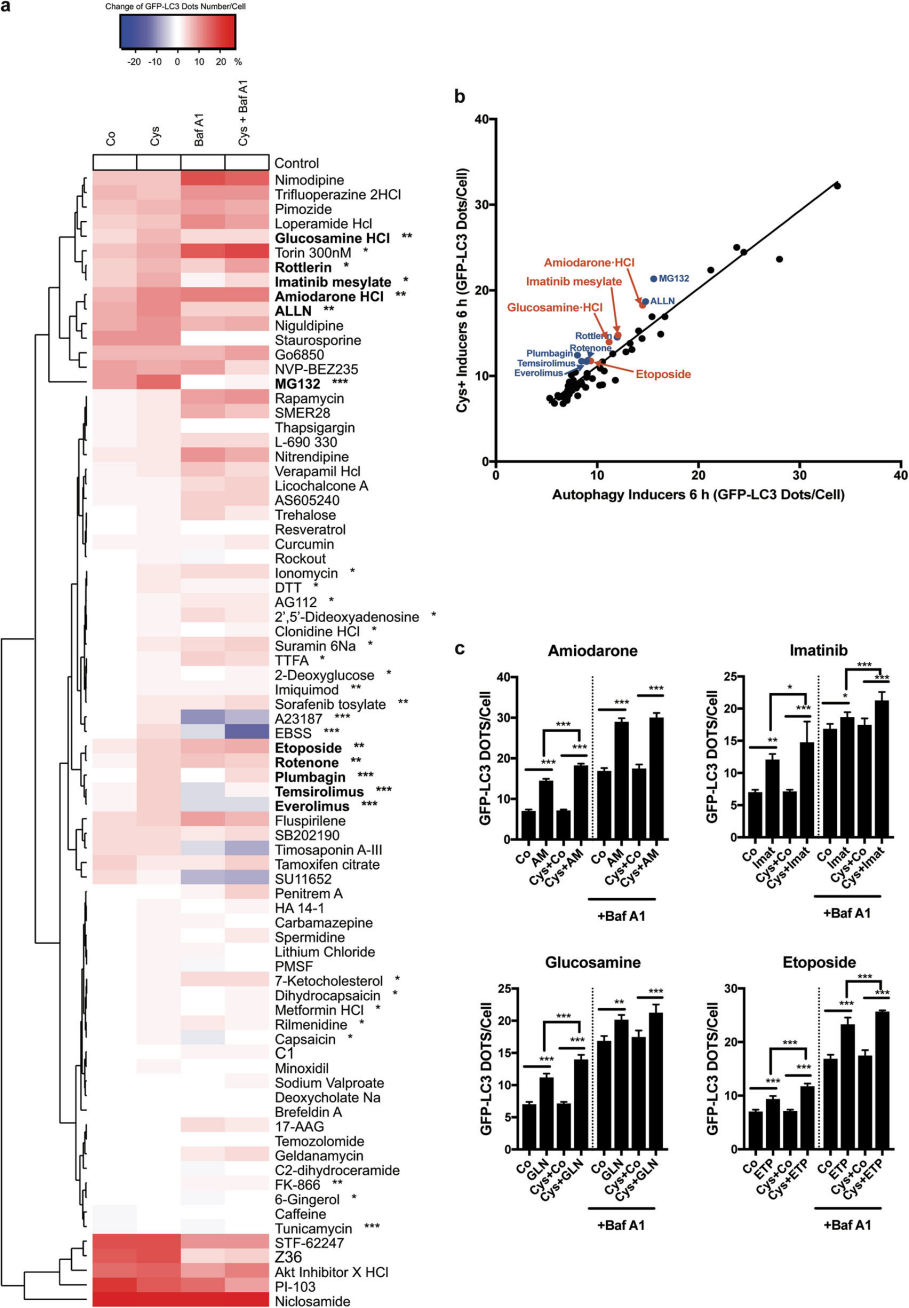
Screening for FDA-approved drugs to stimulate autophagy in a co-treatment with Cysteamine. **a** Heatmap shows change of means of GFP-LC3 dots number/ per cell between each drug and its respective control, significance of additive effects are annotated (*p < 0.05, **p < 0.01, ***p < 0.001), selected positive hits are indicated in bold. The U2OS cells stably expressing GFP-LC3 were pre-treated for 24 h with cysteamine (500 μM) or without as negative control (Co). Then the cells were stimulated with 74 autophagy inducers from SCREEN-WELL® Autophagy library (all at 10 μM), Temsirolimus (500 nM), Everolimus (10 nM), Torin 1 (300 nM) or nutrient free condition (EBSS, Earle’s Balanced Salt Solution) for 6 h. Bafilomycin A1 (Baf A1, 100 nM) was added 2 h before the end of experiment in order to assess the autophagy flux. Hierarchical clustering heatmap was analysed and formed by the R software. **b** Scatter plots showing linear association of the capacity to induce autophagy between single autophagy inducers treatment and the association with the cysteamine (500 μM) pre-treatment. *R*^2^ = 0,9196; *p* < 0.0001; Y = 0,9076*X+2066. In the presence of cysteamine (500 μM), amiodarone (10 μM), imatinib mesylate (10 μM), glucosamine (10 μM), etoposide (10 μM), plumbagin (10 μM), rotenone (10 μM), rottlerin (10 μM), MG132 (10 μM), ALLN (10 μM), everolimus (10 nM), and temsirolimus (500 nM), demonstrate an enhanced capacity in inducing autophagy. **c** Statistic summary of GFP-LC3 dots number per cell from treatment by four FDA-approved drugs amiodarone (10 μM), imatinib mesylate (10 μM), glucosamine (10 μM), etoposide (10 μM), with or without pre-treated cysteamine (500 μM). Bafilomycin A1 (Baf A1, 100 nM) was added 2 h before the end of experiment. Data are expressed as means ± SEM of at least three independent experiments (*p < 0.05, **p < 0.01, ***p < 0.001, compared to untreated cells, Co)

Since EGCG is an over-the-counter agent, yet is not approved as a pharmacological agent, we sought to identify other compounds that might interact with cysteamine to induce autophagy. U2OS cells expressing GFP-LC3 were left untreated or were pre-treated for 24 h with cysteamine according to our standard protocol. Then the cells were stimulated with 74 autophagy inducers from a library of potential autophagy inducers for 6 h in quadruplicate cultures and the number of GFP-LC3 dots per cell were measured. Heatmap recapitulates the strength in the change of the number of dots and the significance of additive effect. Result of hierarchical clustering from the heatmap showed that the presence of the cysteamine have additive effect in inducing autophagy of several drugs among the library (selected positive hits are indicated in bold). (Fig. 4a). We distinguished four main clusters with drug effect varying from very strong to no effect. For example, amiodarone, imatinib and glucosamine cluster in the group of strong drug effect while etoposide, rotenone and plumbagin are grouped in a weak drug effect cluster. We have considered the strength in the change of the number of dots and its significance (p-value). In total, 11 compounds were found to increase the number of GFP-LC3 dots relative to the control, in the presence of cysteamine when compared to untreated cells as showed in the scatter plot (all the positive heats with additive effect are above the diagonal) (Fig. 4b). Among the positive hits, there are four FDA approved drugs (Fig. 4c); we selected two compounds for further studies (namely, the antiarrhythmic Ca^2+^ channel blocker amiodarone and the tyrosine kinase inhibitor imatinib mesylate). Indeed, we have confirmed by pharmacokinetic studies the combined effect of cysteamine and amiodarone (Suppl Fig. 1) or cysteamine and imatinib (Suppl Fig. 2) on classical autophagy parameters, namely GFP-LC3 dots accumulation (Suppl Figs. 1 and 2a, b) and GFP-TFEB translocation from the cytosol to the nucleus (Suppl Figs. 1 and 2c). However, it must be noted that TFEB translocation might be independent of the cysteamine effect since amiodarone has already been described to stimulate TFEB translocation in Hep2G cells^50^.

Quantification by fluorescence microscopy of GFP-LC3 dots number was performed on U2OS GFP-LC3 cells using increasing concentrations of cysteamine (up to 1 mM) alone or in combination with amiodarone (Suppl Fig. 1) or imatinib (Suppl Fig. 2). When given alone, 10 µM of amiodarone induced an accumulation of LC3 dots in a time dependent manner (first column of chessboard plot Suppl Fig. 1a). Amiodarone at 20 µM was excluded from the study because we noticed that the cell number decreases at this concentration. When combined with increasing concentrations of cysteamine, we observed stronger effects on GFP-LC3 puncta. This effect was sustained at later time points (18 and 24 h) when bafilomycin A1 was added 2 h before the end of the experiment (Suppl Fig. 1). Similar effects were observed with imatinib (Suppl Fig. 2), even though the absolute number of GFP-LC3 dots was less than the number obtained with amiodarone. Autophagy stimulation by amiodarone and imatinib was confirmed by their capability to stimulate GFP-TFEB translocation from the cytosol to the nucleus (Suppl Fig. 1c, Suppl Fig. 2c). These results indicated that amiodarone and imatinib stimulate autophagic flux and TFEB activation when combined with cysteamine.

### Effects of autophagy inducers on plasma membrane expression of ∆F508 CFTR

**Fig. 5:**
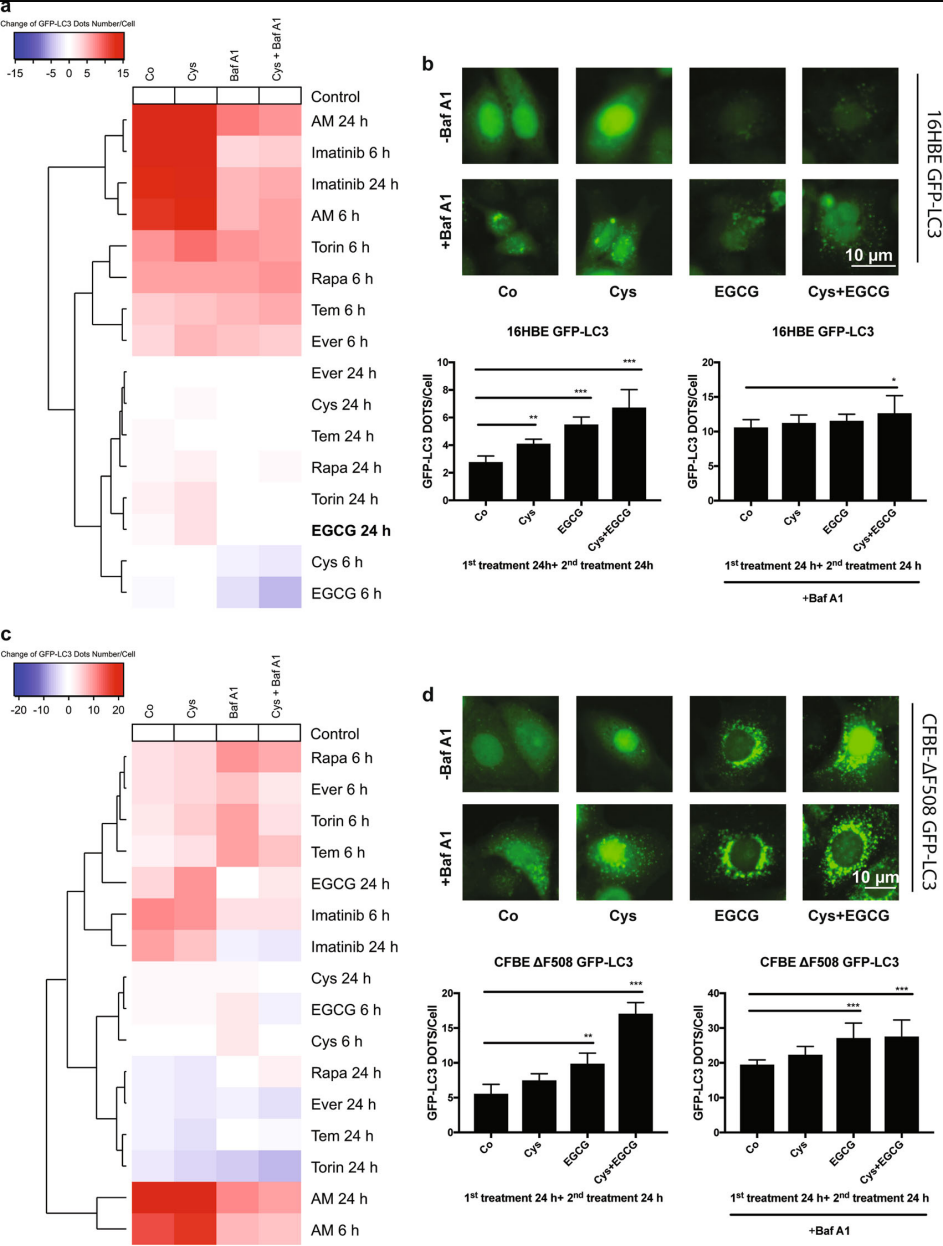
Evaluation of the combined effect of cysteamine and selected drugs to induce autophagy in human epithelial cells. **a**, **c** Hierarchical clustering heatmap shows change of means of GFP-LC3 dots number per cell comparing with its respective control. 16HBE and Cfbe41o- cells stably expressing GFP-LC3 were treated with cysteamine (500 μM) for 24 h and then stimulated for 6 h or 24 h with the indicated drugs. Bafilomycin A1 (Baf A1, 100 nM) was added 2 h before the end of each experiment to evaluate autophagy flux. Hierarchical clustering heatmap was analysed and formed by the R software. (**b**, **d**) Representative images of 16HBE and Cfbe41o- cells stably expressing GFP-LC3 upon exposure to complete medium (Co), cysteamine (Cys, 500 μM), EGCG (160 μM) for 24 h, in the presence or absence of the pre-treatment with cysteamine (Cys, 500 μM) for 24 h. Bafilomycin A1 (Baf A1, 100 nM) was added 2 h before the end of each experiment to evaluate autophagy flux. Data presented in the graph means ± SEM of at least three independent experiments) indicate the GFP-LC3 dots number per cell upon treatment

Next, we tested the effects of cysteamine and selected FDA-approved autophagy inducers on the human epithelial bronchial cell line Cfbe41o- (which is homozygously mutated in CFTR ∆F508) and the corresponding controls 16HBE, which were stably transfected with GFP-LC3 to monitor signs of autophagy (Fig. 5a, c). EGCG and cysteamine induced some accumulation of GFP-LC3 dots in an additive manner in both epithelial cell lines 16 HBE and Cfbe41o- (Fig. 5b, d). Ostensibly, in Fig. 5 the images show that the number of GFP-LC3 dots per cell is higher in CFBE cells (Fig. 5d) than in 16HBE cells (Fig. 5b). Of note, when we generated the epithelial cell lines transfected with the biosensor GFP-LC3 for this study, we observed that the expression level of the biosensor was different. Despite several efforts to homogenize the expression level between the two cell lines, we did not succeed in achieving this goal. As a consequence, basal expression levels of GFP-LC3 are higher in CFBE cells than in 16HBE cells. However, if we compare the relative increase of GFP-LC3 dots number induced by treatment (cysteamine/EGCG)over untreated controls, it appears that the relative increase is similar in 16 HBE cells and CFBE cells.

**Fig. 6:**
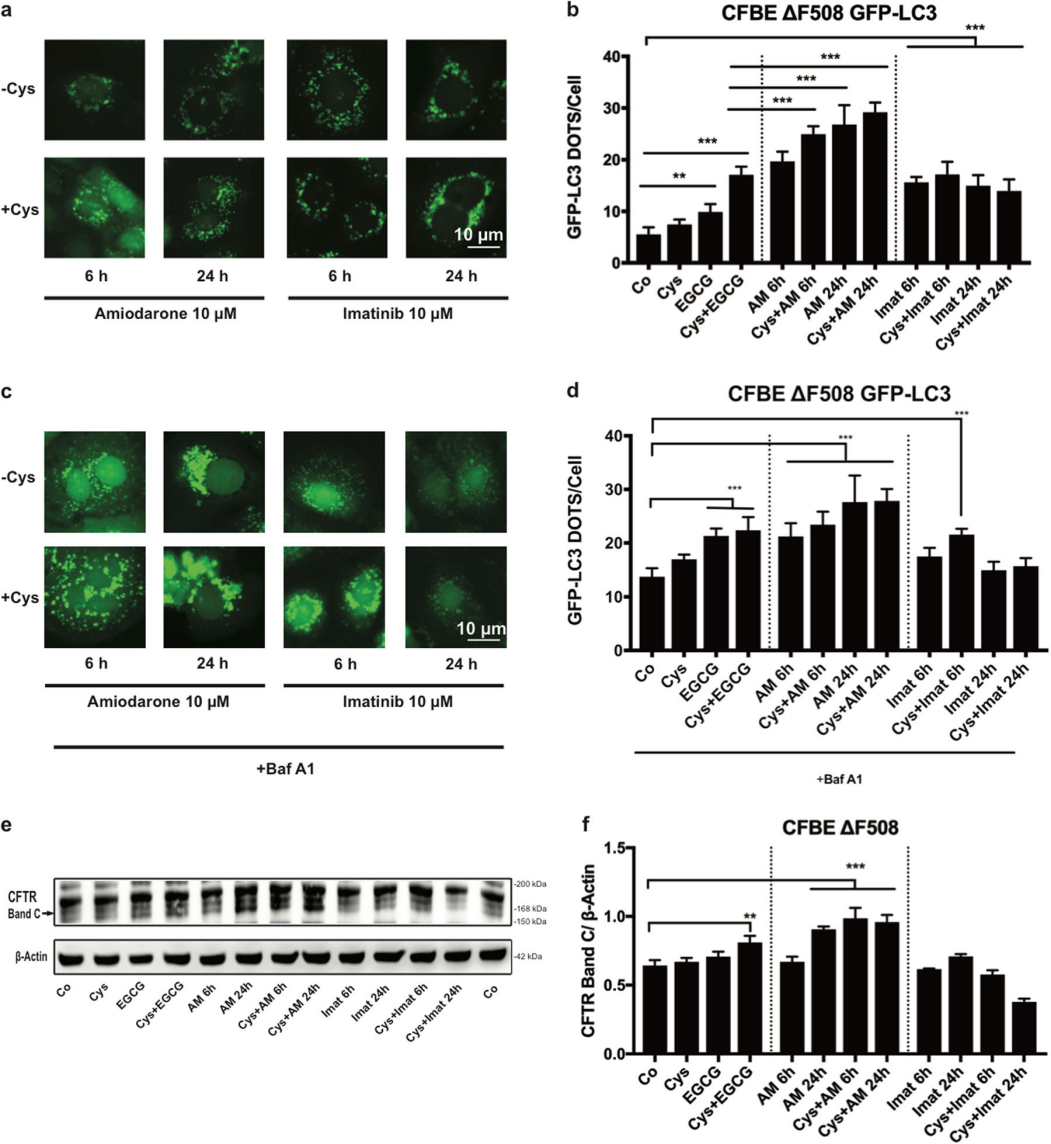
Evaluation of amiodarone induced autophagy and maturation of CFTR in Cfbe41o- human epithelial cells. **a** Representative images of Cfbe41o- cells stably expressing GFP-LC3 treated for 6 h and 24 h with Amiodarone (10 μM), Imatinib (10 μM) with or without 24 h of cysteamine (Cys, 500 μM) pre-treatment. Scale bars equal 10 μm. **b** Data in the graph presented means ± SEM of at least three independent experiments) indicate the GFP-LC3 dots number per cell upon treatment (**p < 0.01, ***p < 0.001, compared to untreated cells, Co). **c**, **d** Representative images and the corresponding bar-plot of Cfbe41 o- cells expressing GFP-LC3 treated as in (**a**) except that bafilomycin A1 was added 2 h before the end of the experiment. **e**, **f** Representative immunoblot of the CFTR band C (which corresponds to the glycosylated form of CFTR). Cfbe41o- cells treated with cysteamine (Cys, 500 μM), EGCG (160 μM), amiodarone (10 μM), imatinib (Imat, 10 μM) alone for 6 h or 24 h except for EGCG (only 24 h) or in combination with 24 h of cysteamine (Cys, 500 μM) pre-treatment. Data are represented as means ± SEM of at least three independent experiments (**p < 0.01, ***p < 0.001 vs. untreated cells)

When amiodarone was added to Cfbe41o- cells, the absolute number of GFP-LC3 dots increased significantly after 6 h of treatment, and this effect could be further increased by cysteamine pre-incubation for amiodarone at 6 h but not for any of the other conditions (Fig. 6a, b). Indeed, at a late time point (24 h) this difference fades. One explanation might be that at longer periods of treatment autophagy induction is already maximal and cannot be increased any more by the combination. Importantly, the combination of cysteamine pre-treatment and EGCG was able to increase the immunoblot-detectable expression of mature plasma-membrane anchored CFTR (band C), and amiodarone (but not imatinib) alone achieved a similar effect, which however was not further enhanced by cysteamine (Fig. 6c, d).

### Concluding remarks

In the present study, we attempted to identify autophagy inducers that might interact with cysteamine to replace EGCG by another compound that would be either more efficient or provide the advantage to be an already approved drug. We have found that similarly to EGCG, amiodarone was able to engage a cooperative interaction with cysteamine to stimulate autophagy in cultured biosensor cell lines. The screening method used here is focused on the identification of molecules capable of restoring the autophagy flux, which has been described as a major event in dictating the fate of ΔF508 CFTR in epithelial cells^51^. Thus, it is based on a surrogate marker, namely the formation of GFP-LC3 puncta (as a method to measure autophagy) rather than CFTR function.

Nonetheless, we identified one agent, amiodarone, that was effective in mediating additive effects on autophagy when combined with cysteamine. Moreover, amiodarone was relatively efficient in stimulating the plasma membrane expression of mature, functional ∆F508 CFTR protein in cultured respiratory epithelial cells. This finding confirms the observation that many different autophagy inducers beyond EGCG may mediate positive effects on ∆F508 CFTR recovery, as this has been also established for α-thymosin^52^ and a panel of inhibitors of the phosphatidylinositol 3-kinase/Akt/mammalian target of rapamycin pathway^53–55^. The exact mechanisms accounting for the ability of amiodarone to induce the re-expression of ∆F508 CFTR are still elusive. Whether this effect relies on its ability to induce autophagy or whether it relies on other cellular pathways that may be influenced by this drug^56,57^ needs further investigation. That said, amiodarone is a widely used antiarrhythmic drug that may cause pulmonary fibrosis as a side effect^58–60^. In spite of this caveat, we are not aware of any case report indicating that amiodarone would have aggravated the lung phenotype of CF patients. Hence, it remains to be evaluated whether amiodarone might be used for the treatment of CF patients, provided that its positive effects on ∆F508 CFTR function would outweigh its side effects on the lung.

## Materials and Methods

The following chemicals and antibodies have been used for this study: cysteamine (M9768; Sigma-Aldrich, St. Louis, MO, USA); EBSS, Earle’s Balanced Salt Solution (E2888, Sigma-Aldrich); epigallocatechin gallate (EGCG, E4143; Sigma-Aldrich); amiodarone (amiodarone hydrochiloride, A8423, Sigma-Aldrich); imatinib (imatinib mesylate, SML1027, Sigma-Aldrich); torin 1 (Tocris Bioscience, Bristol, UK); temsirolimus (PZ0020, Sigma-Aldrich); everolimus (07741, Sigma-Aldrich); bafilomycin A1 (Tocris Bioscience); ENZO library (SCREEN-WELL® Autophagy library, BML-2837-0100, ENZO, USA); anti-beta actin (Abcam 8226, Cambridge, UK); anti-CFTR monoclonal antibody (CF3, ThermoFisher, Waltham, MA, USA).

### Cell culture and transfection

Human Osteosarcoma U2OS cells were cultured at 37 °C and 5% CO_2_ in Dulbecco’s modified Eagle’s medium (DMEM; Life Technologies) supplemented with 100 mM 2-[4-(2-hydroxyethyl) piperazin-1-yl] ethanesulfonic acid (HEPES) buffer, 10% heat-inactivated fetal bovine serum (FBS) (Life Technologies) and 1% penicillin/streptomycin (Life Technologies). Human lung bronchial epithelial cells 16HBE and Cfbe41o- (F508del/F508del-CFTR) were kindly provided by Dr. DC Gruenert (California Pacific Medical Center Research Institute, San Francisco, CA, USA) and cultured as recommended by American Type Culture Collection in Minimum Essential Medium Earle’s salt (200 mM l-glutamine, 10% fetal bovine serum and 100 units/mL penicillin G sodium and 100 μg/mL streptomycin sulfate).

GFP-LC3 stable cell lines were generated by transducing U2OS, 16HBE and Cfbe41o- cells with pre-packaged viral particles expressing recombinant GFP-LC3 (LentiBrite GFP-LC3B Lentiviral Biosensor; Millipore, 17-10193), according to the manufacturer’s instructions. Briefly, cells were plated in a chamber slide and transduced with lentiviral particles at a multiplicity of infection for 24 h. Then, the medium was replaced and cells were visualized after 48 h to monitor transduction efficiency. U2OS cells stably expressing GFP-TFEB were transfected with the pEGFP-N1-TFEB plasmid using the FuGENE® HD transfection reagent protocol. The pEGFP-N1-TFEB plasmid was a gift from Shawn Ferguson (Addgene plasmid #38119).

### High-throughput screening assessment of autophagy by automated fluorescence microscopy

U2OS, 16HBE and Cfbe41o- (F508del/F508del-CFTR) cells, stably expressing GFP-LC3, were seeded into black 96-well plates at 7 × 10^3^ per well. GFP-LC3 U2OS cells and GFP-TFEB U2OS were seeded into 384-well plates (Greiner Bio-One) at 5 × 10^3^ per well. After 24 h, the cells were treated for 24 h with cysteamine then washed with PBS 3× and treated with EGCG or autophagy inducers for 6 h or 24 h, Bafilomycin A1 (Baf A1) was added 2 h before fixation for analysis. Cells were fixed with 4% paraformaldehyde (PFA, w/v in PBS) for 30 min at room temperature or overnight incubation at 4 °C. Nuclei were stained with 10 μM Hoechst 33342 (Molecular Probes-Invitrogen).

Images were acquired using an ImageXpress Micro XLS Widefield High-Content Analysis System operated by the MetaXpress® Image Acquisition and Analysis Software (Molecular Devices, Sunnyvale, CA, USA). Acquisition of Hoechst 33342 and GFP signal was performed by means of a 20× PlanApo objective (Nikon, Tokyo, Japan). A minimum of 9 views fields per well for 96-well plate and 4 view fields per well for 384-well plate were acquired. MetaXpress® was utilised to segment cells into a nuclear area (based on Hoechst 33342 signal), and a cytoplasmic region of interest (ROI). Autophagy was assessed by enumerating GFP -LC3^+^ dots number per cell within each cytoplasmic ROI. In addition, to assess autophagy flux bafilomycin A1 (Baf A1) was added 2 h before the fixation of the cells.

TFEB translocation was assessed for each cell by measuring the ration between cytoplasmic GFP and nuclear GFP intensities with nuclear and cytoplasmic regions defined as described for LC3-GFP expressing cell line.

### Statistical analyses

Data are reported as means ± SD of *n* > 3 replicates and experiments were repeated at least twice yielding similar results. Data were analysed using Prism (GraphPad Software, Inc., La Jolla, CA, USA) and R software (https://www.r-project.org), and statistical significance was assessed by means of two-tailed Student’s *t*-test or ANOVA tests, as appropriate. Hierarchical clustering heatmaps were performed and analysed by R software (Fig. 4a and 5a, c). In the case of additive effect of drug and cysteamine treatment, a linear model was considered: log_10_(Nb of dots) ~ Drug * cysteamine; a *p*-value was associated to the cysteamine coefficient (drug dependent coefficient).

### Immunoblotting

Immunoblotting was performed following standard procedures. Briefly, 10 μg of protein were separated on NuPAGE Novex Bis-Tris 4–12% pre-cast gels (Invitrogen-Life Technologies, Carlsbad, CA, USA) and transferred to Immobilon polyvinylidene difluoride membranes (Merck-Millipore, Darmstadt, Germany). Unspecific binding was reduced by incubating the membranes for 1 h in 0.05% Tween 20 (v/v in TBS) supplemented with 5% w/v bovine serum albumin (Euromedex, Souffelweyersheim, France). Following, membranes were probed with antibodies specific for CFTR (Thermofisher) and beta-Actin (Abcam). Primary antibodies were revealed with species-specific immunoglobulin G conjugated to horseradish peroxidase (Southern Biotech, Birmingham, AL, USA), followed by chemiluminescence analysis with the SuperSignal West Pico reagent by means of an ImageQuant 4000 (GE Healthcare, Little Chalfont, UK).

## Electronic supplementary material

Suppl. Figure 1

Suppl. Figure 2

Supplementary Information
