# Supplementary figures and images for: Evaluation of autophagy inducers in epithelial cells carrying the ΔF508 mutation of the cystic fibrosis transmembrane conductance regulator CFTR
Source: Cell Death Dis. 2018 Feb 7;9(2):191. doi: 10.1038/s41419-017-0235-9 (PMC5833759; doi:10.1038/s41419-017-0235-9)

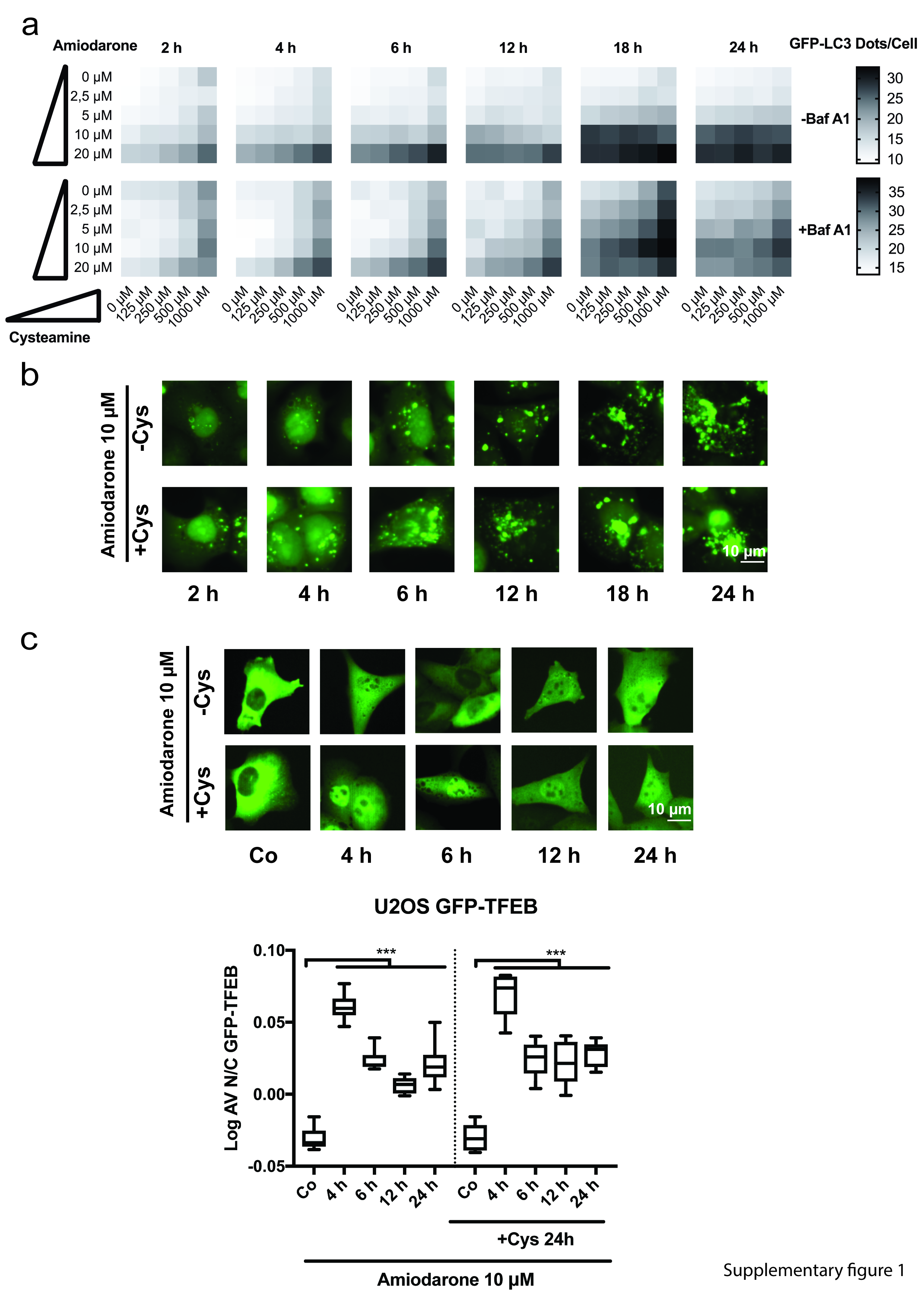

Supplement: Supplementary file 1 — Suppl. Figure 1 [file 41419_2017_235_MOESM1_ESM.tif]

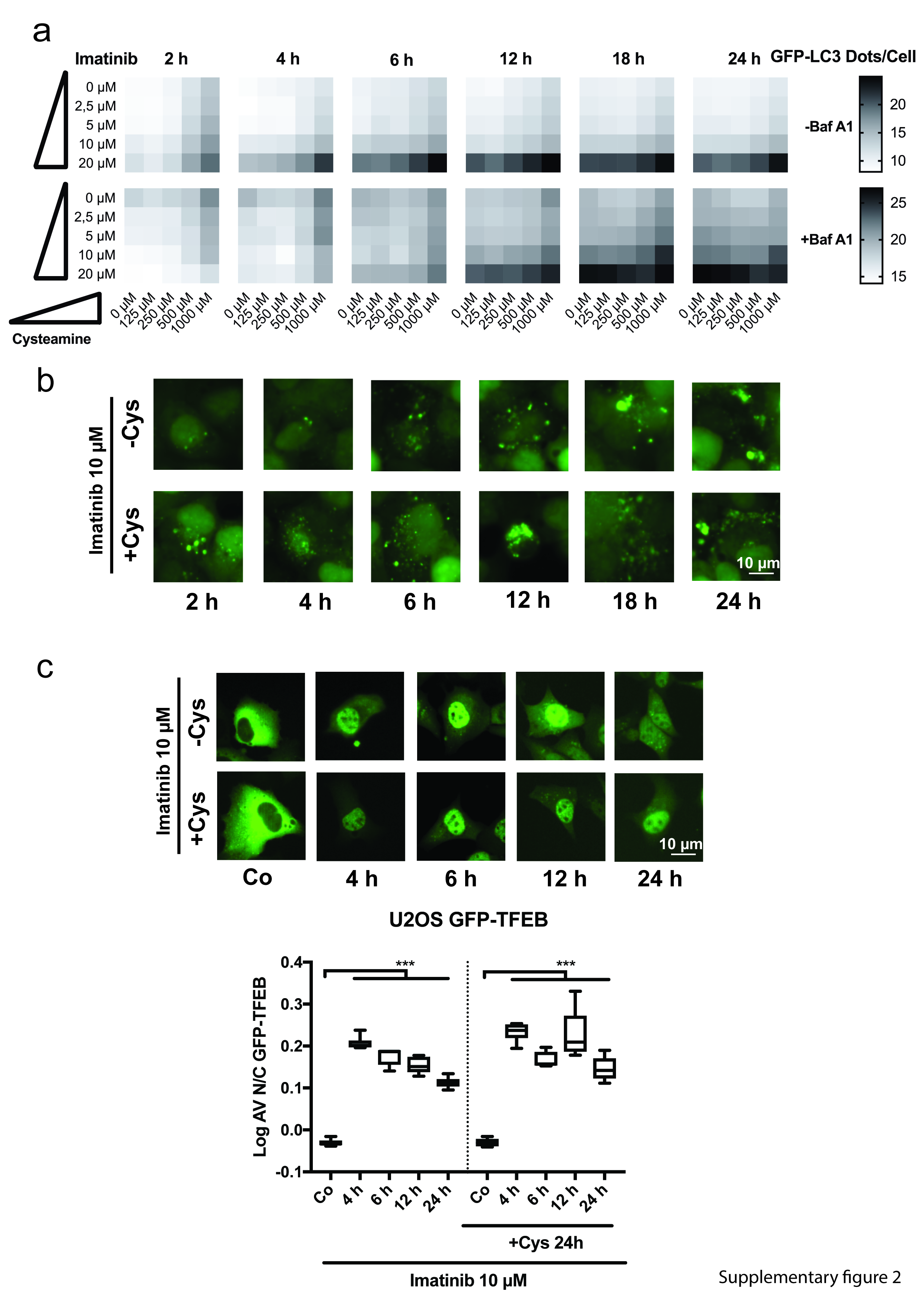

Supplement: Supplementary file 2 — Suppl. Figure 2 [file 41419_2017_235_MOESM2_ESM.tif]
