# Supplementary material for: Evaluation of autophagy inducers in epithelial cells carrying the ΔF508 mutation of the cystic fibrosis transmembrane conductance regulator CFTR
Source: Cell Death Dis. 2018 Feb 7;9(2):191. doi: 10.1038/s41419-017-0235-9 (PMC5833759; doi:10.1038/s41419-017-0235-9)
Supplement: Supplementary file 3 — Supplementary Information [file 41419_2017_235_MOESM3_ESM.docx]

**Supplementary Figure Legends**

**Supplementary Figure 1. Cooperative effect between Cysteamine and Amiodarone in inducing autophagy**

(a) Chessboard graph summarising the pharmacokinetic additive effect of 24 h pre-treatment of cysteamine using 5 different concentrations (0, 125, 250, 500, 1000 μM) followed by washout by PBS and incubation with amiodarone (AM) at 5 different concentrations (0, 2.5, 5, 10, 20 μM) from 2 to 24 h in GFP-LC3 U2OS cells. Evaluation of autophagy flux was done by a high content microscopic screening evaluating the formation of GFP-LC3^+^ dots per cell. Bafilomycin A1 (Baf A1, 100 nM) was added 2 h before the end of the experiment. (b) Representative images of U2OS GFP-LC3 cells upon exposure to amiodarone (10 μM) from 2 to 24 h in the presence /absence of cysteamine (500 μM, 24 h) pre-treatment. (c) Representative images of GFP-TFEB U2OS cells upon exposure to complete medium (Co) and amiodarone (10 μM) from 2 to 24 h in the condition of presence /absence of 24 h cysteamine (500 μM) pre-treatment. The graph shows the average ratio between GFP-TFEB florescence intensity in the nucleus to the cytoplasm (means ± SD, n=3; ***p < 0.001 compared to untreated cells).

**Supplementary Figure 2. Cooperative effect between Cysteamine and Imatinib in inducing autophagy**

(a) Chessboard graph summarising the pharmacokinetic additive effect of 24 h pre-treatment of cysteamine using 5 different concentrations (0, 125, 250, 500, 1000 μM) followed by washout by PBS and incubation with imatinib at 5 different concentrations (0, 2.5, 5, 10, 20 μM) from 2 to 24 h in GFP-LC3 U2OS cells. Evaluation of autophagy flux was performed by a high content screening microscopic assessing the number of GFP-LC3^+^ dots per cell. Bafilomycin A1 (Baf A1, 100 nM) was added 2 h before the end of the experiment. (b) Representative images of U2OS GFP-LC3 cells upon exposure to imatinib (10 μM) from 2 to 24 h in the presence /absence of cysteamine (500 μM, 24h) pre-treatment. (c) Representative images of GFP-TFEB U2OS cells upon exposure to complete medium (Co) and imatinib (10 μM) from 2 to 24 h in the presence or absence of cysteamine (500 μM, 24 h) pre-treatment. The graph shows the average ratio between GFP-TFEB florescence intensity in the nucleus to the cytoplasm (means ± SD, n = 3; ***p < 0.001 compared to untreated cells).
